# Genome-Wide Technologies to Study RNA–Chromatin Interactions

**DOI:** 10.3390/ncrna6020020

**Published:** 2020-05-27

**Authors:** Masaki Kato, Piero Carninci

**Affiliations:** Laboratory for Transcriptome Technology, RIKEN Center for Integrative Medical Sciences, Yokohama, Kanagawa 230-0045, Japan

**Keywords:** long non-coding RNA, chromatin-associated RNA, RNA-chromatin interaction, gene regulation

## Abstract

An increasing number of studies have revealed that long non-coding RNAs (lncRNAs) play important roles in gene regulation and nuclear organization. Although the mechanisms are still largely unknown, many lncRNAs have been shown to interact with chromatin. Thus, one approach to understanding the function of these lncRNAs is to identify their sites of genomic interaction. Hybridization capture methods using oligonucleotide probes have been used for years to study chromatin-associated RNA. Recently, several groups have developed novel methods based on proximity ligation to investigate RNA–chromatin interactions at a genome-wide scale. This review discusses these technologies and highlights their advantages and disadvantages for the consideration of potential users.

## 1. Introduction

Chromatin-associated RNAs (chrRNAs) have important roles in gene regulation, genomic organization, and chromatin structure maintenance. Although the majority of chrRNAs are nascent transcripts attached to chromatin by RNA polymerase II (Pol II) [1], chrRNAs also include many long non-coding RNAs (lncRNAs) that function in specific regions of the genome. The most famous and well-studied example is *XIST* lncRNA, responsible for X chromosome inactivation in mammals [2,3]. *XIST* lncRNA is expressed from only one of the two X chromosomes. It coats the X chromosome and recruits multiple complexes including epigenetic modifiers that establish gene silencing and formation of facultative heterochromatin.

ChrRNAs (or lncRNAs) are generally categorized into two groups based on their mode of action. LncRNAs that regulate the expression of nearby genes are defined as *cis*-acting. However, it is often hard to distinguish the mature RNA molecules that regulate gene expression from transcribed lncRNAs involved in gene regulation independently of their RNA sequence. For example, the *Airn* lncRNA gene, which overlaps with *Igf2r* in antisense orientation in the mammalian genome, is required for silencing the paternal genes in the *Igf2r* cluster. It has been reported that transcription through the *Igf2r* promoter, rather than the sequence of the mature *Airn* RNA, leads to *Igf2r* silencing [4]. Subsequent study has shown that the *Airn* RNA molecules recruit repressive chromatin modifiers to distant, non-overlapped genes like *Slc22a2*, *Slc22a3,* and *Pde10a* to cause silencing [5]. Thus, the *Airn* lncRNA gene has two ways to repress gene expression in the *Igf2r* cluster. Although *XIST* is considered to be a *cis*-acting lncRNA, it can act over long genomic distances through travel of *XIST* RNA to distal regions of the X chromosome. Thus, the mode of action of *cis*-acting lncRNAs can be further subclassified.

The other group of chrRNAs, which regulate gene expression on a genome-wide scale, consists of *trans*-acting lncRNAs. *HOTAIR* is one of the *trans*-acting lncRNAs reported. *HOTAIR*, is expressed from the *HOXC* locus and has been reported to silence *HOXD* genes by recruiting the repressive chromatin modifiers PRC2, LSD1, and CoREST/REST [6,7,8]. However, later studies raised doubts regarding this initial model. It has been reported that overexpression of *HOTAIR* in the MDA-MD-231 breast cancer cell line represses several genes independently of PRC2 [9]. In addition, it is still controversial whether *HOTAIR trans*-regulates *HOXD* cluster genes owing to inconsistencies in the results of studies using *Hotair* mutant mice [10,11,12,13].

The functions of chrRNAs (or lncRNAs) have been reviewed comprehensively elsewhere [14,15]. This review focuses on recent technologies to capture RNA–chromatin interactions. Early hybridization capture methods using oligonucleotide probes (so-called “one-to-all” methods because they involve a single target RNA), such as Chromatin Isolation by RNA Purification (ChIRP), Capture Hybridization Analysis of RNA Targets (CHART), and RAP-DNA (RNA antisense purification), have been used extensively for a long time. Using these methods, *XIST* RNA was found to spread on the X chromosome at an initial stage of X-chromosome inactivation [16,17]. Many sites of lncRNA interaction, including *MALAT1* and *NEAT1*, were also identified by using one-to-all methods [18]. However, these methods might be suitable only for lncRNAs for which the mechanism is known, but for which it has not been possible to identify novel regulators. The FANTOM5 consortium showed that approximately 19,000 human lncRNAs likely to be functional [19] and that ~35% of those found in human dermal fibroblasts (HDFs) are nuclear enriched [20]. In addition, in some cases these one-to-all methods suffer from high background signals [21], preventing researchers from clearly interpreting their results and producing unambiguous results. For these reasons, non-biased, high-throughput methods to non-selectively capture all nuclear RNAs are required. Recently, four groups developed related “all-to-all” methods based on the use of proximity ligation to capture RNA–chromatin interactions on a genome-wide scale. We compare these new technologies to each other and to the one-to-all methods.

## 2. Technologies to Capture RNA–Chromatin Interactions

### 2.1. One-to-All Methods

**Figure 1:**
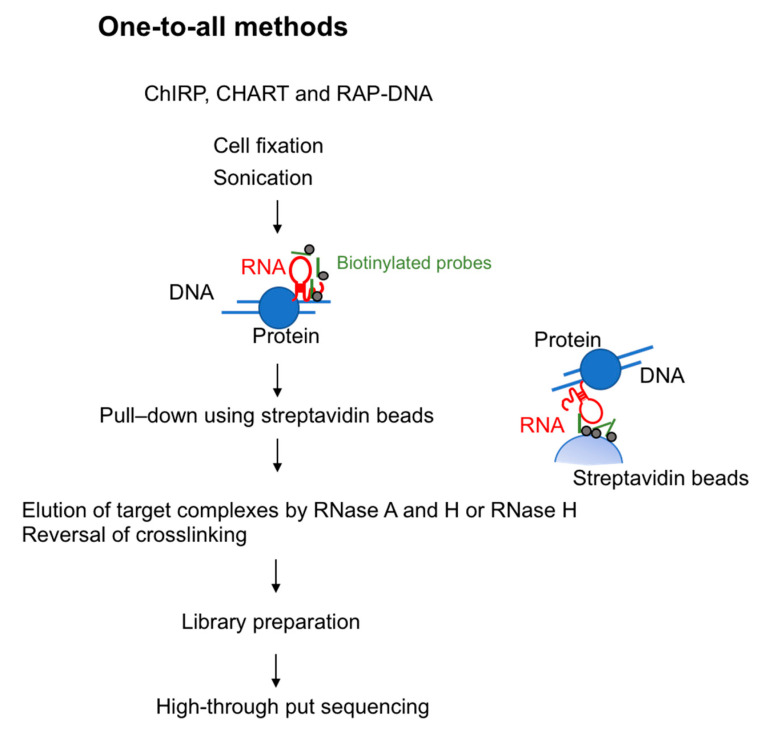
Overview of one-to-all methods to capture RNA-interacting genomic loci. Chromatin Isolation by RNA Purification (ChIRP), Capture Hybridization Analysis of RNA Targets (CHART), and RAP-DNA (RNA antisense purification) use biotinylated probes for the target RNA. After the chromatin fraction is pulled down by the use of streptavidin beads, RNase is used to elute genomic DNA. The resulting library is prepared for high-through put sequencing.

To investigate lncRNA binding sites on chromatin, in 2011 two groups developed methods using biotinylated DNA probes. These methods are termed ChIRP-seq (chromatin isolation by RNA purification) [22] and CHART-seq (capture hybridization analysis of RNA targets) [23]. In 2013, RAP-DNA (RNA antisense purification) was developed: This method uses longer antisense RNA probes to increase specificity [16]. These three methods are similar in that all use hybridization-based strategies. Specifically, they use biotin-conjugated probes that hybridize with target RNA and pull down the chromatin fraction associated with that RNA species (Figure 1).

#### 2.1.1. Chromatin Isolation by RNA Purification (ChIRP-seq)

ChIRP-seq uses 20-mer biotin-conjugated DNA oligonucleotide probes to recognize the target lncRNA. Cells are crosslinked by glutaraldehyde or formaldehyde to stabilize RNA-chromatin interaction, sonicated for DNA fragmentation and then hybridized with the probes. To ensure specificity, multiple probes are designed and split into two separate pools (referred to as the “odd” and “even” pools) that identify common targets. After the reversal of crosslinking, genomic DNA is analyzed by high-throughput sequencing to identify genomic regions associated with the lncRNAs. To test the reliability of ChIRP-seq, the authors performed ChIRP-seq targeting *roX2*, a gene involved in dosage compensation in *Drosophila* and associated with many regions on the X chromosome in *Drosophila* cells [22]. They also isolated *HOTAIR*-associated DNA, demonstrating that *HOTAIR* lncRNA preferentially associated with regions containing a GA-rich DNA motif [22]. ChIRP-seq is the most commonly used one-to-all method because the DNA probes can be designed without prior knowledge of the target RNA structure or functional domain.

#### 2.1.2. Capture Hybridization Analysis of RNA Targets (CHART-seq)

CHART-seq, developed by Simon et al., is conceptually similar to ChIRP-seq [23]. This protocol uses 25-mer desthiobiotin-conjugated DNA oligonucleotide probes. An RNase H sensitivity assay is used to identify regions in the target lncRNA that are accessible to the probes. These genomic regions are enriched followed by high-throughput DNA sequencing to identify the loci bound by the targeted lncRNAs. To minimize background, RNase H is used to elute RNA–chromatin complexes bound to the oligonucleotide probes. Any binding of genomic DNA or proteins to the probes and streptavidin magnetic beads is non-specific; thus, in theory only RNAs bound specifically to the probes would be eluted. CHART-seq was first used to investigate the function of *roX2* in *Drosophila* [23], and was later used to study the *Xist* genome-wide binding regions [17].

#### 2.1.3. RNA Antisense Purification (RAP-DNA)

RAP-DNA is another method that can be used to identify chromatin regions that interact with RNAs [16]. RAP-DNA uses a set of 120-nt antisense RNA probes that have high binding affinity to the target lncRNA (for example, pool of 120-nt probes tiled every 15 nucleotides across the entire *Xist* transcript (~17 kb)). The genomic regions are isolated followed by high-throughput DNA sequencing. Using RAP-DNA, the authors showed that *Xist* RNA is initially transferred to chromosome regions of the X chromosome that have high contact frequencies with the *Xist* transcription site as determined by genome-wide Chromatin Conformation Capture (Hi-C), and then gradually spreads to other accessible locations [16].

### 2.2. All-to-All Methods

**Figure 2:**
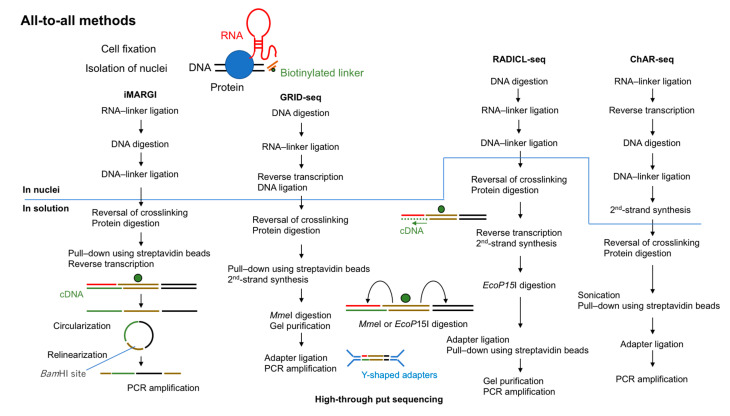
Overview of all-to-all methods to capture RNA-interacting genomic loci. All four methods (MARGI (mapping RNA–genome interactions), GRID-seq (global RNA interaction with DNA sequencing), RADICL-seq (RNA and DNA interacting complexes ligated and sequenced), and ChAR-seq (chromatin-associated RNA sequencing)) use fixed cells and biotinylated 5′-adenylated linkers. The steps above the line are performed in nuclei and those below the line are performed in solution.

In 2017, two groups reported the first “all-to-all” methods to detect RNA–chromatin interactions genome-wide. These methods were termed MARGI (mapping RNA–genome interactions) and GRID-seq (global RNA interaction with DNA sequencing) [24,25]. In 2018, ChAR-seq (chromatin-associated RNA sequencing) was reported [26]. Very recently (in 2020), RADICL-seq (RNA and DNA interacting complexes ligated and sequenced) was developed [27]. All of these methods are based on proximity ligations using a bivalent linker that can ligate to RNA at one end and to digested DNA at the other end (Figure 2).

#### 2.2.1. MARGI and iMARGI

MARGI was invented by Sridhar et al. [24] in 2017. A derived protocol reported in 2019, iMARGI, reduced the required number of input cells [28]. The original MARGI protocol uses approximately 4 × 10^8^ cells fixed in 1% formaldehyde. After nuclear isolation, all proteins are biotinylated using iodoacetyl PEG2 biotin (IPB) in preparation for subsequent purification using streptavidin beads. This step can ease the numerous buffer changes required in MARGI. In iMARGI, the ligation steps are performed in situ instead of on streptavidin beads. This modification may contribute to the lower number of input cells required for iMARGI (~5 × 10^6^) owing to less diffusion of RNA molecules or to less non-specific ligation in nuclei. Both MARGI and iMARGI use a biotinylated bivalent DNA linker to ligate RNA and DNA in formaldehyde-fixed nuclei. In MARGI, streptavidin beads require to be blocked with D-biotin before RNA ligation. Because both proteins and the linker are biotinylated this is done to prevent free linkers from binding to streptavidin beads. The 5′ end of this linker is pre-adenylated with Mth RNA ligase. T4 RNA ligase 2, truncated KQ is used for RNA ligation, which ligates the pre-adenylated 5′ end of the linker to the 3′ OH end of the RNA. The double-stranded end of the linker ligates with T4 DNA ligase to the digested and end prepared genomic DNA (digested by *Hae*III for MARGI and by *Alu*I for iMARGI). It is critical to keep track of the directionality of the linker to identify the RNA or DNA end for subsequent data analysis. As discussed later (Section 2.2.2 and Section 3.1.4), in GRID-seq and RADICL-seq the entire linker must be sequenced to identify the source of each end of the ligation product. To overcome this issue, in MARGI the ligation product is circularized after reverse transcription and then relinearized by cutting at the *Bam*HI site in the middle of linker sequence. The halves of the digested linker are designed to be parts of the two PCR primers which are usable for the Illumina sequencing platform. The relinearized library can be directly amplified by PCR without further linker ligation.

#### 2.2.2. GRID-seq

GRID-seq uses a biotinylated bivalent linker to ligate RNA and DNA in situ in nuclei double-fixed in formaldehyde and disuccinimidyl glutarate. Approximately 2 × 10^6^ mammalian cells or 1 × 10^7^ *Drosophila* cells are required. RNA ligation is performed, and then the ligated RNA is converted to cDNA by using one of the DNA strands in the linker as a primer. The other end of the linker is ligated to *Alu*I-digested genomic DNA. Although GRID-seq uses a bivalent linker structurally similar to that used for MARGI, the linker for GRID-seq consists of single-stranded RNA (rather than DNA) that is pre-adenylated at the 5′ end. T4 RNA ligase 2, truncated KQ is used for RNA ligation, which can ligate pre-adenylated 5′end of DNA or RNA to the 3′ OH end of the RNA. However, the authors have not reported which linker with pre-adenylated DNA or RNA could be ligated to RNA more efficiently and why they used the linker with pre-adenylated RNA [25,29]. After reversal of crosslinking and protein digestion, linker-ligated products are selectively purified by biotin. *Mme*I is then used to cut 18–20 bases away from its recognition sites in the linker to generate paired-end tags (cDNA (18–20 bp)—linker-DNA—genomic DNA (18–20 bp)). After separation by PAGE, digested products of the correct size are purified from the gel and then ligated to the Y-shaped sequencing adapters, which preserve the directionality of the library for PCR amplification.

#### 2.2.3. ChAR-seq

ChAR-seq is another method to capture RNA–chromatin interactions based on proximity ligation. First, RNA is ligated to a biotinylated bivalent linker. After second-strand synthesis using one of the DNA strands in the linker as a primer, genomic DNA is digested by *Dpn*II and DNA ligation reactions are performed. Unlike in GRID-seq, the ligated molecules in ChAR-seq are sonicated to generate smaller fragments for library construction. ChAR-seq has been tested initially with *Drosophila* cells. Later the authors reported the use of human embryonic stem cells (hESCs), human retinal pigmented epithelial cells (hTERT RPE-1) and nuclei isolated from *Xenopus laevis* embryos in their protocol paper although they did not provide any data [30]. Random ligations may be more common in mammalian cells than in *Drosophila* cells because the genome size of mammals is much larger than that of *Drosophila*. If so, mammalian cells will require more high-throughput sequencing reads to generate usable sequence reads.

#### 2.2.4. RADICL-seq

RADICL-seq is the latest all-to-all sequencing method and was published in 2020 [27]. Despite its conceptual similarity to other all-to-all methods, the authors pointed out several key differences that improve its performance relative to other methods for studying RNA–chromatin interactions. The authors used ~2 × 10^6^ mammalian cells (mouse ES cells (mESCs) and mouse oligodendrocyte progenitor cells (mOPCs)) fixed in formaldehyde (1% or 2%). One end of the linker is single-stranded DNA that is pre-adenylated at the 5′ end for RNA ligation and the other end is double-stranded DNA that is ligated to genomic DNA ends digested by DNase I. Similar to the GRID-seq linker, the RADICL-seq linker contains two restriction enzyme recognition sites (in this case, for *Eco*P15I) that direct cutting within the RNA and DNA ends of the ligation product to generate tags of 25–27 bp each. After separation by PAGE, digested products of the correct size are purified from the gel. The Y-shaped sequencing adapters are then ligated to the biotin-purified fragments for library construction. One unique feature of RADICL-seq is the use of RNase H treatment to reduce abundant nascent transcripts. As an additional control, actinomycin D, which inhibits Pol II, is used before cell fixation to stop Pol II transcription and thus eliminate nascent transcripts.

## 3. Comparison of Four All-to-All Methods

All four all-to-all methods described above are conceptually similar because they are based on proximity ligations; however, they have some differences in experimental conditions and data processing. In brief, they differ in four main technical aspects: (1) number of cells required, (2) crosslinking conditions, (3) method used to digest genomic DNA, and (4) use of long reads versus reads trimmed by restriction enzyme digestion (Table 1). We discuss each of these in the following sections.

### 3.1. Experimental Conditions for All-to-All Methods

#### 3.1.1. Cell Systems

MARGI and RADICL-seq have been tested only with mammalian cells [24,27,28], whereas ChAR-seq was initially tested only with *Drosophila* cells. GRID-seq has been tested with both *Drosophila* and mammalian cells. Because mESCs were analyzed using both GRID-seq and RADICL-seq, these methods can be compared directly. The original MARGI protocol uses ~4 × 10^8^ cells (human embryonic kidney (HEK) and H9 (human ESC)), which is a fairly large number of cells compared with other methods. Development of iMARGI reduced the required cell quantity to a more treatable number, i.e., ~5 × 10^6^ human foreskin fibroblasts (HFFs). GRID-seq used ~2 × 10^6^ mammalian cells (human MDA-MB-231 (breast cancer cells), MM.1S (multiple myeloma cells) and mouse ESC) or 1 × 10^7^ *Drosophila* cells (S2). ChAR-seq was successfully used with 1-1.5 × 10^8^ *Drosophila* cells (CME-W1-cl8+ and Kc167). RADICL-seq was successfully performed with 2 × 10^6^ mammalian cells (mouse ESCs and OPCs). Thus, GRID-seq and RADICL-seq could be achieved with only 2 × 10^6^ cells, whereas MARGI and ChAR-seq require higher numbers of cells (Table 1). It will be possible to achieve with less number of cells after refining of their protocol. However, although sequencing libraries can be made from the cell number indicated or less for each method, the result is more directed by the endogenous expression level of the RNAs. Thus, the amount of initial material required will depend on the expression level of the target RNA to be investigated. In particular, lncRNAs tend to have lower expression than coding RNAs [31]. More over RNA extraction issue has to be dealt with even though the expression level of the RNA is high. For example, it has been reported that extensive needle sharing or heating of cell lysate during RNA extraction could improve the extraction of *NEAT1* lncRNA, which forms a paraspeckle nuclear bodies [32]. When choosing the number of cells, one should take the expression level or the solubility of the RNA of interest into account to obtain enough tags for the RNA–chromatin interactions.

#### 3.1.2. Crosslinking Conditions

Among the four methods, all except GRID-seq use formaldehyde to crosslink cells. Testing of RADICL-seq with 1% and 2% formaldehyde fixation gave highly comparable results, so 1% is now commonly used. The GRID-seq method uses a higher concentration of formaldehyde (3%) in the fixation step and additional disuccinimidyl glutarate (DSG), a strong protein–protein crosslinker. Although the authors did not show the data, they mentioned that they found more small RNAs when using 3% formaldehyde + DSG than with weaker crosslinking conditions (i.e., 1% formaldehyde) [29]. The RADICL-seq procedure also includes a condition called “non-protein mediated” in which proteinase K digestion and reversal of crosslinking are done before the RNA ligation reaction. Under this condition, RNA–DNA interactions would be captured only if the binding is direct and not mediated by proteins. Overall, the fixation step is critical for all protocols. Mechanisms of RNA localization are varied (e.g., direct interaction to DNA by R-loops and triple helixes or indirect interaction via proteins or RNA); thus, the choice of fixation method might depend on how the RNAs interact with chromatin and should be further explored during future technology development.

#### 3.1.3. Genomic DNA Digestion

All four protocols begin with the nucleus isolation step by using a lysis buffer containing non-ionic detergent (0.2% NP-40), after which the nuclei are permeabilized by SDS. Except for RADICL-seq, which uses DNase I to digest the genomic DNA, the protocols use restriction enzymes (MARGI, *Hae*III (iMARGI: AluI); GRID-seq, *Alu*I; ChAR-seq, *Dpn*II).

Bonetti et al. [27] found that the genomic coverage of DNA regions identified by RADICL-seq was higher than that by GRID-seq data. This could have resulted from the stronger crosslinking condition in GRID-seq and the use of *Alu*I for DNA digestion, which introduces biases in the genome in particular for Alu elements (the enzyme is named *Alu*I because it cleaves Alu repeats very frequently and Alu elements are located in gene-rich regions) [33]. In contrast, it has been shown that DNase I causes intrinsic DNA-cutting biases towards DNase-seq [34]. Thus, digestion conditions for each cell type should be tested before library construction. It is important to keep in mind that every digestion method has its own bias.

#### 3.1.4. Long Reads vs. Reads Trimmed by Restriction Enzyme Digestion

After RNA and DNA ligation to the bivalent linker, the methods differ in how the ligation products are treated. In MARGI, the ligated molecules are circularized for library construction. GRID-seq and RADICL-seq use a linker that contains two recognition sites for type II and III restriction enzymes, respectively (*Mme*I and *Eco*P15I, respectively) to digest the ligated products into fixed-size fragments. In ChAR-seq, the ligation products are sonicated to obtain smaller fragments for library construction. One of advantages of iMARGI is that longer sequencing reads can be obtained. On the other hand, in GRID-seq and RADICL-seq the entire linker must be sequenced to identify which end of the sequence corresponds to RNA and which corresponds to DNA. To obtain fixed-size fragments can be sequenced, GRID-seq uses *Mme*I, which cuts both the RNA and DNA ends to generate tags of 18–20 bp each, and only these 18–20 bp will be used for mapping. RADICL-seq instead uses *Eco*P15I, which digests 25–27 bp away from its recognition sites. RADICL-seq therefore results in longer reads for mapping and about three times as many uniquely mapped reads than GRID-seq (45% vs. 14% of mappable reads to the mouse genome). The restriction-enzyme-based methods such as GRID-seq and RADICL-seq ensure that all reads contain both RNA and DNA sequences; in contrast, MARGI and ChAR-seq do not produce fixed-size fragments and require more sequencing reads to obtain comparable numbers of RNA and DNA interactions. Future technology development should consider methods to achieve even longer reads to further complete the RNA/DNA interactome with more broadly repeated sequences of the genome, including retrotransposons.

### 3.2. Data Processing

#### 3.2.1. Sequencing

In the original MARGI report, the library is sequenced with 100 cycles of paired-end sequencing on an Illumina platform. The authors suggest obtaining 300 million or more read pairs [35]. For GRID-seq, a single-end 100-cycle kit was used for sequencing on an Illumina HiSeq 2500 sequencer. The authors sequenced at an average depth of 160 million reads per replicate for mouse ESC libraries [29]. For ChAR-seq, the libraries are typically sequenced with 152 single-read cycles or paired-end 150 reads. The authors sequenced more than 400 million reads for *Drosophila* cell libraries [26,30].

For RADICL-seq, ~120 million reads were sequenced using the 150-cycle Illumina kit [27]. Usable reads are very limited for any of these protocols after mapping. For example, in the case of ChAR-seq the authors have multiple steps to extract usable reads: (1) Removal of PCR duplicates, (2) extraction of reads containing linker adapters, (3) splitting of RNA and DNA reads, (4) alignment, and (5) removal of rRNA. At the end, only 10% of the total reads are useful for downstream analysis [26]. GRID-seq of mammalian cells returned 26–39% unique mapped read pairs, and RADICL-seq (using 1% FA) returned 13–19%, although the investigators might have used different alignment tools for each other. As a result, the required sequencing depths depend on the question researchers want to address. With MARGI, the interaction between *XIST* and the X chromosome and the interaction between *MALAT1* and the *NEAT1* gene locus could be identified with ~30 million total read pairs [29], although *XIST* and *NEAT1* are known to be highly expressed lncRNAs.

#### 3.2.2. Dealing with Background

One of the important challenges during data analysis is to differentiate specific from non-specific RNA–chromatin interactions. In principle, proximity-based methods can capture significant amounts of nascent transcript because transcribing or newly transcribed RNAs are very close to their transcription sites. In addition, transcribed RNAs might interact with all accessible chromatin regions in a non-specific manner; for instance, RNA molecules diffusing in the nucleus may be captured during library construction. In a test of GRID-seq, mixed *Drosophila* and mammalian cells were used as the input to estimate false positives; the results showed that 6.8% of the RNAs linked to *Drosophila* DNAs were of mammalian origin [25]. The specificity of ChAR-seq was tested using spike-in RNAs, and the results showed less than 0.5% non-specific ligation [26]. Both GRID-seq and RADICL-seq include data normalization methods for statistical analysis to correct for background. The correction method used in GRID-seq was inspired by the strategy developed for processing Hi-C data [36]. Hi-C is a high-throughput technology to measure the probability of contact between different chromosome loci; however, Hi-C data have many biases due to the ligation efficiencies of different fragment sizes, frequencies of restriction enzyme recognition sites in the genome and GC content. Thus, several normalization methods have been developed for the Hi-C method. In brief, GRID-seq uses *trans*-acting reads of mRNAs to estimate background and normalize the data [25]. The authors provided the pipeline for data analysis (GridTools) [29]. RADICL-seq uses the one-sided cumulative binomial test to detect significant RNA-chromatin interactions and the Benjamini–Hochberg multiple-testing correction to correct for the false discovery rate. After the test, “significant interactions” were extracted to use their downstream analysis [27]. After background correction, only 1.5% of *trans-*interaction reads remained in the mESC data of GRID-seq. Similarly, in RADICL-seq only 0.8% of the total significant dataset was attributed to *trans* interaction. Thus, for GRID-seq and RADICL-seq, the majority of *trans* interactions in the raw data are considered background. The MARGI and ChAR-seq methods do not include any statistical methods to deal with the background for all RNA–chromatin interactions at once. Instead, statistical tests are performed according to the biological question being addressed [24,26,28,37]. In summary, it is very important to define what to consider as background. Even when RNAs are localized to their usual site of interaction, what is their biological meaning, especially in the case of nascent RNAs? Although many interactions may happen, it is hard to distinguish which ones are meaningful in terms of gene regulation.

## 4. Comparison of All-to-All Methods with One-to-All Methods

The major advantage of using all-to-all methods is that we can identify de novo RNA-chromatin interactions. For example, a GRID-seq analysis provided information about the strength of enhancers, showing that RNAs interact more frequently at a super-enhancer locus than at a typical enhancer locus [25], and a RADICL-seq study demonstrated that repeat elements were differentially engaged in specific chromatin interactions in different cell types [27].

The major limitation of all-to-all methods is the requirement for a large number of sequencing reads to detect RNA species with low expression and to generate interpretable interaction data. To validate the specificity of the detected biological interactions, each all-to-all method should be compared with one or more one-to-all methods using several RNAs as positive controls. *roX2* is a well-known lncRNA involved in dosage compensation in *Drosophila*. Interestingly, in a comparison of the raw data from ChIRP-seq, CHART-seq and GRID-seq, the percentage of total raw *roX2* DNA reads interacting with the X chromosome was the highest with GRID-seq (~70%), moderate with ChIRP-seq (25–30%) and lowest with CHART-seq (10%) [25]. These data suggest that specificity of one-to-all methods is lower than that of all-to-all methods. We need to reevaluate the lncRNA-interacting sites detected by one-to-all methods by comparing the results with those from all-to-all methods (Table 2).

## 5. Conclusions

Here, we have reviewed several one-to-all and all-to-all technologies used to study RNA–chromatin interactions. In particular, we have argued that the proximity-based all-to-all methods are more powerful for understanding the role of chromatin-associated RNAs and genome organization. Zhou et al. also pointed out that Hi-C was a main technology to study genome organization before developing of the proximity-based all-to-all methods to capture RNA–chromatin interactions. The all-to-all methods have more advantages to study cell-type-specific genomic interactions because RNAs play roles for cell-type-specific gene regulation even though DNA-DNA contacts detected by Hi-C are not varied [29]. As discussed above, the first major limitation of all-to-all methods is the need to distinguish true interactions from background introduced by nascent transcripts. To overcome this problem, we need to develop further strategies. For example, the developers of RADICL-seq introduced RNase H treatment into their protocol to reduce nascent transcripts or actinomycin D treatment before cell fixation to stop Pol II transcription. The second limitation is the sensitivity to low-abundance RNAs. One strategy that should be considered is combining all-to-all methods with a capture strategy for specific RNAs or antibody pull-down to enrich RNAs associated with molecules of interest. For example, using an anti-Pol II antibody may enrich promoter-enhancer RNA (eRNA) interactions, and antibodies against CCCTC-binding factor or some subunits of cohesin may help reveal enrichment of transcription-associated RNAs within topologically associating domain boundaries.

In conclusion, these technologies to study RNA–chromatin interactions have great potential to enable exploration of chrRNAs and identify their functions in gene regulation and genome organization. By building on these current protocols, researchers may develop more advanced protocols, perhaps even capable of analyzing single cells. Recently RIC-seq (RNA in situ conformation sequencing), which is also a technology based on proximity ligation was reported [40]. This technology can generate three-dimensional interaction maps of RNA to study regulatory roles of RNA. More novel lncRNAs will be discovered and their biological functions will be investigated. Additional methods of capturing RNA–chromatin interactions, including one-to-all methods, will always be welcome to help us identify the function of lncRNAs.

## Figures and Tables

**Table 1:** Comparison of conditions for MARGI, GRID-seq, ChAR-seq, and RADICL-seq.

Technical Aspect	MARGI (iMARGI) *	GRID-Seq	ChAR-Seq	RADICL-Seq
Crosslinking condition	1% formaldehyde	DSG + 3% formaldehyde	3% formaldehyde	1% formaldehyde
Genomic digestion	*Hae*III (*Alu*I) *	*Alu*I	*Dpn*II	DNase I
Reduction of nascent transcripts	none	GridTools	none	RNase H Actinomycin D One-sided cumulative binomial test
Carrier to pellet nuclei and wash between enzymatic steps	Streptavidin beads	none	none	SPRI beads
Length of RNA and DNA tags	long	18-20 bp	long	25–25 bp
Cell types tested	human HEK293 H9 (hESC) (Human foreskin fibroblast [HFF]) *	human MDA-MB-231 (breast cancer cells) MM.1S (multiple myeloma cells) mouse mESC *Drosophila* S2 cells	*Drosophila* CME-W1-cl8+ cells (male) Kc167 (female)	moues mESC OPC
Cell number	4 × 10^8^ cells (5 × 10^6^ cells) *	2 × 10^6^ cells (mammalian) 1 × 10^7^ cells (*Drosophila*)	1–1.5 × 10^8^ cells (*Drosophila*) 1–1.5 × 10^7^ cells (human)	2 × 10^6^ cells

* Items marked with asterisks pertain to iMARGI.

**Table 2:** RNAs analyzed by both all-to-all methods and one-to-all methods.

	RAP-DNA	ChIRP-seq	CHART-seq
GRID-seq	*Malat1* (mESC) [38]	*roX2* (S2) [22]	*roX2* (S2) [23] *MALAT1* (GRID: MDA-MB-231, CHART: MCF7 [18])
ChAR-seq		*roX1, roX2* (ChIRP: S2 [22], ChAR: CME-W1-cl8+)	
RADICL-seq	*Malat1* (mESC) [38]	*Rn7SK* (mESC) [39]	
